# Children and Snakebite: Snake Venom Effects on Adult and Paediatric Plasma

**DOI:** 10.3390/toxins15020158

**Published:** 2023-02-14

**Authors:** Christina N. Zdenek, Caroline F. B. Rodrigues, Lachlan A. Bourke, Anita Mitico Tanaka-Azevedo, Paul Monagle, Bryan G. Fry

**Affiliations:** 1 Venom Evolution Lab, School of Biological Sciences, The University of Queensland, St. Lucia, QLD 4072, Australia; 2 Laboratório de Herpetologia, Instituto Butantan, São Paulo 05508-040, SP, Brazil; 3 Programa de Pós-Graduação Interunidades Em Biotecnologia, USP, IPT e Instituto Butantan, São Paulo 05508-040, SP, Brazil; 4 Department of Paediatrics, University of Melbourne, Parkville, VIC 3010, Australia; 5 Haematology Research, Murdoch children’s Research Institute, Flemington Rd., Parkville, VIC 3052, Australia; 6 Department of Clinical Haematology, Royal Children’s Hospital, Flemington Rd., Parkville, VIC 3052, Australia; 7 Kids Cancer Centre, Sydney Children’s Hospital, High St., Randwick, NSW 2031, Australia

**Keywords:** paediatrics, pediatrics, VICC, hemostasis, child, development, blood coagulation, envenomation, elapid, viper

## Abstract

Snakebite is a globally neglected tropical disease, with coagulation disturbances being the primary pathology of many deadly snake venoms. Age-related differences in human plasma have been abundantly reported, yet the effect that these differences pose regarding snakebite is largely unknown. We tested for differences in coagulotoxic effects (via clotting time) of multiple snake venoms upon healthy human adult (18+) and paediatric (median 3.3 years old) plasma in vivo and compared these effects to the time it takes the plasmas to clot without the addition of venom (the spontaneous clotting time). We tested venoms from 15 medically significant snake species (from 13 genera) from around the world with various mechanisms of coagulotoxic actions, across the three broad categories of procoagulant, pseudo-procoagulant, and anticoagulant, to identify any differences between the two plasmas in their relative pathophysiological vulnerability to snakebite. One procoagulant venom (*Daboia russelii*, Russell’s Viper) produced significantly greater potency on paediatric plasma compared with adult plasma. In contrast, the two anticoagulant venoms (*Pseudechis australis*, Mulga Snake; and *Bitis cornuta*, Many-horned Adder) were significantly more potent on adult plasma. All other procoagulant venoms and all pseudo-procoagulant venoms displayed similar potency across both plasmas. Our preliminary results may inform future studies on the effect of snake venoms upon plasmas from different age demographics and hope to reduce the burden of snakebite upon society.

## 1. Introduction

Snakebite is a serious health issue with high public impact, particularly in rural, impoverished regions of the tropics [1,2,3]. In June 2017 the World Health Organization re-recognised snakebite in the highest category (A) of Neglected Tropical Diseases, and global efforts are underway to reduce the burden of snakebite on communities [4,5,6]. Snakebite envenomation can affect any biological target reachable by the bloodstream and thus results in complex medical emergencies [7]. The primary pathologies leading to death from snakebite are neurotoxicity (affecting skeletal muscles) and coagulotoxicity (disrupting normal blood clotting) [8], making these syndromes of snakebite particularly important to understand and for which to have clinical management plans.

A particularly difficult snakebite syndrome to treat is coagulopathy, i.e., the disruption of haemostasis. The main reason for this is due to the complexity of the haemostatic system and because, unlike post-synaptic neurotoxicity quickly recovering after antivenom administration [9], venom effects to the blood system are not immediately reversed by antivenom. This is due to a considerable time (14+ h) required between venom neutralisation of the toxins and the resynthesis of clotting factors to recover from their depletion [10]. To achieve haemostasis, a biochemical system of checks-and-balances within the blood occurs via primary (formation of ‘platelet plug’ on the vessel wall) and secondary (‘coagulation cascade’) haemostasis. Proteins within the cascade are referred to as factors, most of which are produced in the liver, and remain inactive (as zymogens) in the blood until required and activated (into proteolytic enzymes) typically by either tissue factor or collagen [11]. This series of inactive proteins (zymogens) become activated during injury (even microscopic internal injury) and, under different scenarios, either activate or suppress other factors in the cascade in a tightly balanced fashion, with various negative and positive feedback loops. The endogenous forms of these coagulation factors have been mimicked (in conformation and activity) [12,13] and weaponised [14,15] by venoms of many venomous snakes, resulting in these limbless predators effectively over-dosing the victim to catastrophically disrupt the body’s normal haemostasis.

Research into children coagulation—and even more so the relative susceptibility to snakebite—has been impeded due to smaller quantities in blood samples, greater difficulty in acquiring samples, and greater variability between samples (related to the gestational age and postnatal age of the infant) [16]. However, to improve clinical care and patient outcome in paediatric snakebite cases, it is important to better understand any pathophysiological differences induced by deadly venoms upon this vulnerable demographic.

Children are often more vulnerable than adults to snakebite for multiple reasons. Their inquisitive nature and lack of personal restraint and/or awareness may render them at greater risk to envenomation [17]. For example, children in north-eastern South Africa being twice as likely to be envenomated as adults [18]. In addition to frequency, severity of envenomation is often greater in children due to distances walked after the bite before treatment [17]. Furthermore, being physically smaller is likely to increase the severity of the medical emergency from snakebite envenomation due to relatively higher venom concentrations, i.e., a lower dilution effect in children during envenomation. These relatively high venom concentrations are evident in: thromboembolic strokes in a 2-year-old toddler bitten by *Pseudonaja textilis* (Eastern Brown Snake) [19]; the compartmental syndrome observed as a common complication in over half of envenomed paediatric patients in Costa Rica [20]; more severe cytotoxicity in children than adults from South Africa [21,22]; higher rates of necrosis, ecchymycosis, and pulmonary edema in children in Turkey [23]; several forms of rattlesnake-induced coagulation disturbances being more common in paediatric patients than adult patients [24]; and particularly poor outcomes in patients ≤5 years [25].

Beyond body size differences, age-related biochemical differences can also affect snakebite prognosis. Children have important quantitative and functional differences in their blood compared with adults, a concept termed Developmental Haemostasis [26]. Differences between paediatric and adult plasma can exist in (1) the concentration and function of procoagulant and anticoagulant blood components [27,28], (2) the turnover rate (i.e., resynthesis of consumed factors) of these components, and (3) the overall ability of the hemostatic system to generate and regulate two key components thrombin and plasmin [16]. Specifically, most coagulation proteins, such as prothrombin levels and thrombin generation, are lower in children [29], with the exception of FVIII and FXIII not changing significantly with age [28]. Age-related differences in coagulation factors appears to continue beyond childhood, as was observed via thromboelastography (TEG) in a study of 60 healthy male and 60 healthy female patients aged 19–87 [30]. Age-related differences in the levels of coagulation factors, and subsequent possible alteration on standard coagulation tests, may have serious implications for diagnosis and medical treatment, including for snakebite.

Procoagulant venoms mostly lead to incoagulability (inability to clot due to consumption of fibrinogen) [10] and occasionally cause stroke (cerebral infarcts) in adults [31,32] and children [33]. Children may be more susceptible to stroke due to a likely higher venom-to-blood ratio in their small body relative to adults. This situation aligns more closely with the effects observed in small mammalian prey: the highly concentrated procoagulant snake toxins within a small blood volume produce a clot which travels to the brain where it is large enough to occlude a small blood vessel [34,35,36,37].

Most studies that describe differences in snakebite across multiple age demographics rely on retrospective clinical data of patients. Such on-the-ground comparisons are valuable in that the whole-of-body effect of envenomation is considered. However, classic coagulation tests on envenomated patients may be insensitive due to positive feedback loops of the coagulation cascade and the long length of time post-envenomation that these tests are performed [38]. In addition, this type of interrogation cannot exclude confounding factors such as venom dose injected, duration until treatment, management of the snakebite (e.g., extent of physical movement post-bite), and variable health pre-bite. Thus, in vitro work can provide a valuable all-else-equal comparison of the susceptibility of different demographics to snakebite.

There is a need to reduce the burden of snakebite upon society [6] and a need for further study regarding the implications of developmental hemostasis [39]. However, literature directly comparing snake venom effects on paediatric plasma compared to that of adults is, to our knowledge, absent, with studies instead investigating plasma from envenomated patients after the damage has already occurred. Such studies cannot track venom action in real time or control for multiple factors affecting envenomation, such as venom dose or time since envenomation. In this study we report the direct effect of a wide range (15) of medically significant snake venoms upon paediatric plasma, which was pooled from 22 healthy children (ages 1.3–4.9 years old; median 3.3), compared to pooled adult plasma (unknown number of individuals; 292 mL; 18+ years old). Our results provide insights into the relative pathophysiological susceptibility of adult and paediatric plasma to coagulotoxic snake venoms from around the world. 

## 2. Results and Discussion

We used a coagulation analyser to measure the venom-induced clotting times of adult and paediatric plasma by 15 coagulotoxic snake species from around the world (Table 1). We added snake venom to healthy plasma and observed the immediate and direct effects on two different human plasma types (adult and paediatric) compared to the control. In this way we controlled for the venom concentration, the age of patients, and time since envenomation. While valuable for different purposes, testing plasma from envenomed patients leads to insensitivity to classic coagulation tests [38,40].

**Figure 1:**
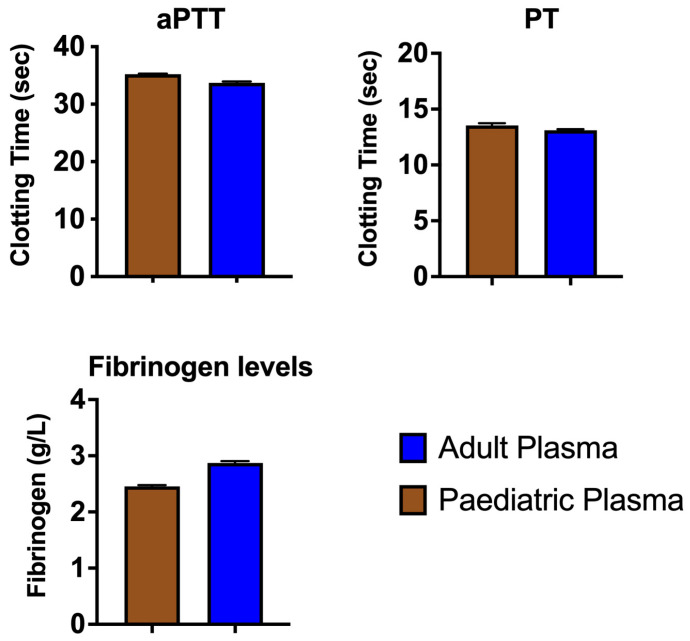
Means of *n* = 3 ± SD of coagulation tests on adult and paediatric plasma batches used in this study. These standard coagulation tests provide a baseline comparison to other works. Methods are detailed in Table 2. The nonparametric Kolmogorov–Smirnov test revealed no significant differences between plasma types.

The results from three standard coagulation assays on our healthy adult and paediatric plasma batches (Figure 1) aligned with expectations from the literature (e.g., lower fibrinogen levels in paediatric plasma) [55]. When comparing the average AUC values (Area Under the Curve) for 8-point dilution curves ran in triplicate, which ranged in venom concentration from 0.05 µg/mL to 20 µg/mL, we found that *Pseudonaja textilis* venom was the most potent venom on both plasmas, with clotting times at the 20 µg/mL venom concentration being 5.3–6.1 s. Remarkably, this approaches Claussian clotting time (3.2 s), a clotting time produced by adding excess thrombin to plasma to rapidly convert all available fibrinogen into fibrin [56]. All venoms affected both plasma types similarly (no significant differences), except for three venoms, one of which is procoagulant in nature (*Daboia russelii*), and two of which are anticoagulant (*Pseudechis australis* and *Bitis cornuta*) (Table 2). *Daboia russelii* venom clotted paediatric plasma significantly quicker than adult plasma, indicating a greater drop-for-drop susceptibility of paediatric plasma to this venom. In contrast, *P. australis* and *B. cornuta* venoms were significantly less potent on paediatric plasma relative to adult plasma. 

The venom from *Daboia russelii* was included because this species is a highly medically significant snake that occurs throughout 10 South Asian countries. In combination with its sister species, *D. siamensis*, these snakes are the leading cause of fatal snakebite in India, Bangladesh, Pakistan, Sri Lanka, Burma and Thailand [57,58]. Despite being sluggish and reclusive, flooding events and habitat invasion by humans bring *D. russelii* in contact with humans, particularly in plantations, which make up a significant proportion of ﻿the human-dominated rural landscape in places such as Karnataka, India [59]. As a result, unfortunately the human-snakebite conflict regarding *D. russelii* is acute. However, the relative effect of *D. russelii* venom in children has been largely unknown, with one case report of a 10-year-old girl who developed gross myoglobinuria (occurrence of myoglobin (a protein in muscles) in urine; associated with myotoxicity) [60].

**Figure 2:**
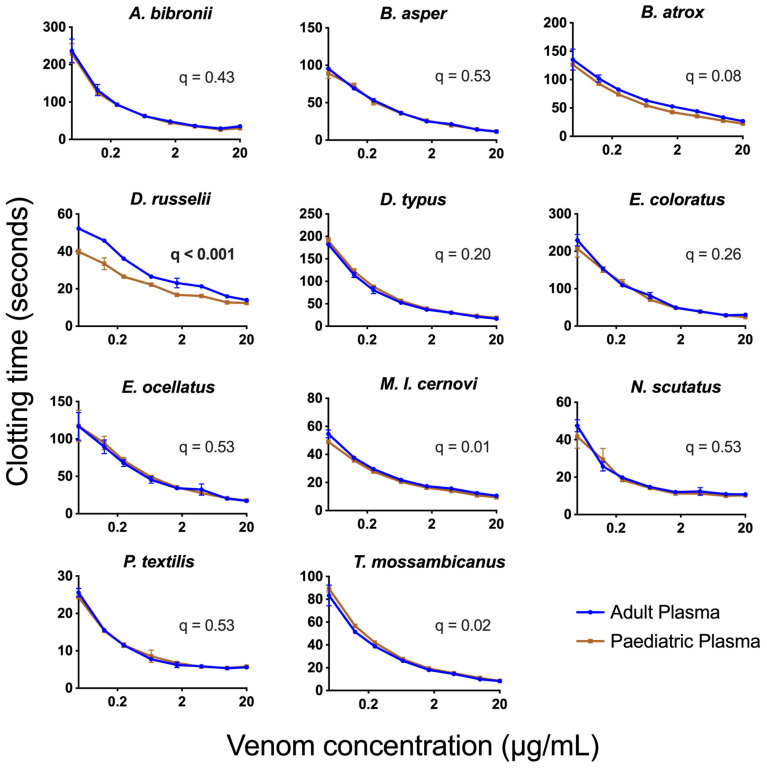
Eight-point venom-dilution curves (0.05–20 µg/mL) comparing the venom effects upon adult and paediatric plasma, in log view. ‘Procoagulant venom assay’ methodology used is detailed in Table 2. Quicker clotting times indicate greater coagulotoxicity. The negative control for adult plasma was 675.0 ± 19.4 s and for paediatric plasma was 629.7 ± 16.1 s (not shown). Different *y*-axis scales were used to assist plasma comparisons within each subfigure. A 1% False Discovery Threshold of unpaired *t*-tests of AUC values (Table 2) revealed q-values shown (bolded = significant). Data points are means of *n* = 3 replicates ± SE error bars.

We observed a significantly greater potency of *D. russelii* venom upon paediatric plasma compared with adult plasma in vitro (Figure 2). This finding suggests that a physiological difference between these two plasmas affects the binding or enzymatic kinetics of the venom toxins. The exact cause of this differential potency of *D. russelii* venom upon the two plasma types is unclear, but some hypotheses are herein proposed. *Daboia russelii* venom is known to activate FX (via PIIId SVMPs) and FV (via serine proteases) [61,62,63]. Both of these zymogens have been shown (functionally and immunologically) to occur at lower levels in paediatric plasma compared with adults [28,64]. As such, it may be the case that the venom more quickly activates the fewer zymogen targets available, resulting in the greater observed potency in paediatric plasma. Conversely, lower levels of molecular targets could theoretically result in a reduced venom effect. Another possibility is post-translational modification differences in the zymogens [65] may alter toxin binding. Consequently, future work should (1) add an excess of FX and FV zymogens to paediatric plasmas to observe if this alters *D. russelii* venom potency and (2) measure binding constants of venom fractions (individual toxins) to FX and FV zymogens.

Another biological aspect of plasma that theoretically may affect the potency of snake venom is the availability of phospholipids in the blood. Phospholipids are negatively charged ions released in vivo by activated platelets that are a required substrate on which some coagulation complexes form during coagulation [50]. Despite platelets being absent in the platelet-poor plasma used in the present study, small amounts (estimated 3%) of phospholipid are still present in plasma [66], and phospholipids were also added into the assay (Table 2) in the same quantity as standard coagulation assays in hospital settings. Regarding the venoms used in this study and their dependence on endogenous phospholipids for their coagulotoxicity, our previous work showed that *D. russelii* venom ﻿is unusually highly dependent upon phospholipid for the activation of Factor X [49], which is not mirrored by species within the genera *Macrovipera*, *Oxyuranus*, or *Pseudonaja* [49,67]. However, no differences in phospholipid levels have been observed between paediatric and adult plasma [68], suggesting that phospholipid levels do not explain the differential potency of *D. russelii* venom upon the two plasmas. The observed difference may therefore be related to the mechanism of procoagulant action and requires future work to elucidate.

**Figure 3:**
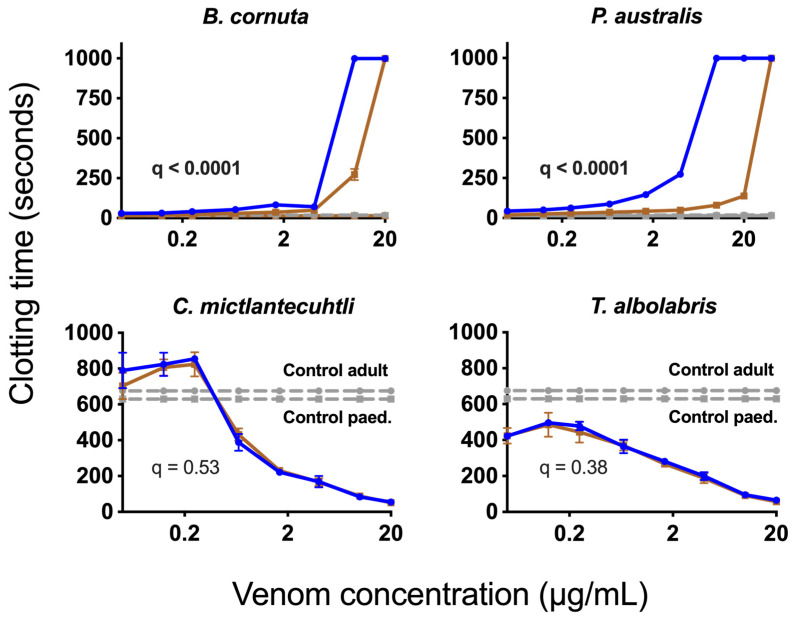
Eight-point venom dilution curves (0.05–20 µg/mL) comparing the venom effects upon paediatric (brown line) and adult (blue line) plasma, of anticoagulant venoms using the ‘anticoagulant venom assay’ (top row) and pseudo-procoagulant venoms using the ‘procoagulant venom assay’ (bottom row) in Table 2. Grey lines indicate negative control values without venom. Top row: slower clotting times (higher y-value) indicate greater coagulotoxic potency in the anticoagulant assay. Note that one additional dilution point (40 µg/mL) was added to the *P. australis* curve to determine if paediatric plasma would reach maximum clotting time (999 sec) of the machine. Bottom row: quicker clotting times (lower y-value) indicate greater coagulotoxic potency for the pseudo-procoagulant assay. A 1% False Discovery Threshold of unpaired *t*-tests of AUC values (Table 2) revealed q-values shown (bolded = significant). Data points are means of *n* = 3 replicates ± SE error bars.

**Figure 4:**
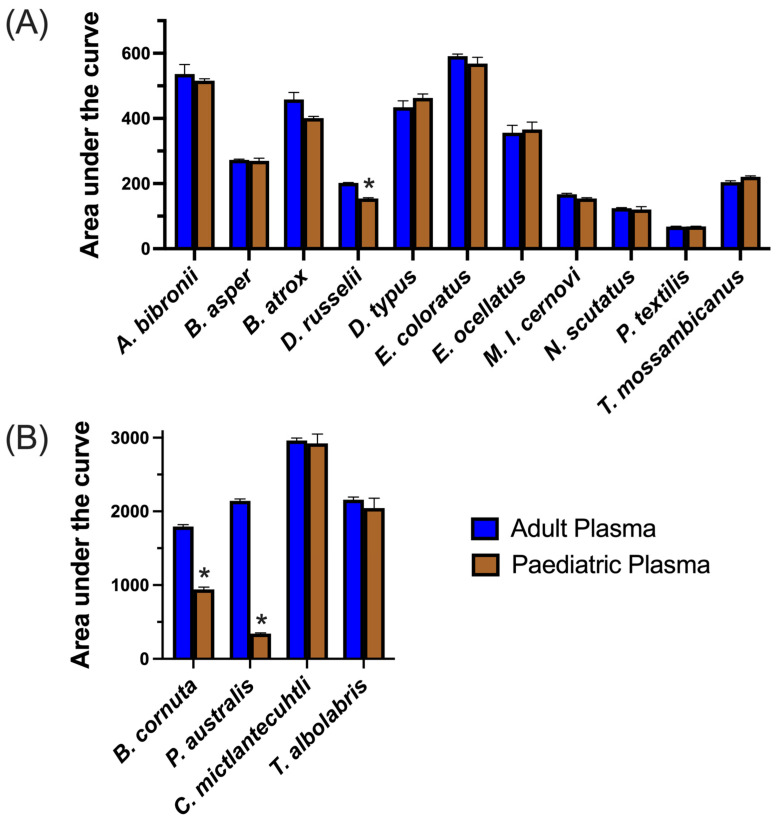
Relative potency of (**A**) 11 procoagulant snake venoms, plus (**B**) two anticoagulant and two pseudo-procoagulant venoms across adult and paediatric plasma. Area Under the Curve (AUC) values were derived from eight-point dilution curves run (Figure 2 and Figure 3) in triplicate for each plasma for each venom. Smaller AUC values for procoagulant and pseudo-procoagulant venoms indicate greater potency, while larger AUC values for anticoagulant venoms (*P. australis* and *B. cornuta*) indicate greater potency. Data are AUC means derived from 8-pt dilution curves run in triplicate, with error bars representing standard deviations. A 1% False Discovery Threshold of unpaired *t*-tests of AUC plasma values revealed q-values shown in Table 2 (* = significant, i.e., *p* < 0.05).

In contrast to the higher potency of *D. russelii* venom upon paediatric plasma, we observed a decreased potency of both anticoagulant venoms (*P. australis* and *B. cornuta*) on paediatric plasma relative to adult plasma (Figure 3). The relative potency of all venoms is compared in Figure 4. The mechanism of anticoagulant action by *P. australis* and *B. cornuta* appears to be the binding to and inhibition of the prothrombinase complex via PLA_2_s [51,69]. The endogenous activity of PLA_2_s (hydrolysis of phospholipids) was excluded as a possible mechanism for anticoagulation by these venoms because a removal of phospholipids from the prothrombinase-inhibition assay (used in that study and the present study) only resulted in slightly delayed clotting times, compared with complete inhibition of clotting times in the presence of venom [42]. Given that prothrombin (the target of prothrombinase) levels are lower in children than adults [70], there may be less substrate upon which the venom toxins can indirectly affect, thus possibly limiting its potency. However, the zymogen targets of *D. russelii* venom (FX and FV) also occur at lower levels in paediatric plasma, yet that venom was *more* potent upon paediatric plasma. Furthermore, no significant differences were observed with several other venoms (*D. typus, N. scutatus, O. scutellatus,* and *P. textilis*) that activate prothrombin [71], suggesting that lower zymogen levels in paediatric plasma does not always (or necessarily) affect venom potency. 

Another possible explanation for the relatively decreased potency of anticoagulant venoms (*P. australis* and *B. cornuta*) on paediatric plasma is the decreased potential in infants and children to convert prothrombin to thrombin [27], perhaps making the venom effects upon prothrombinase less impactful to the system. The prothrombinase complex in children may also have an altered structure such as altered post-translational modifications. Regardless, the mechanism behind the greater potency of *D. russelii* venom upon paediatric plasma is unknown. Unusually, systemic myotoxicity always occurs in adults but rarely children bitten by *Daboia russelii* [60]. The reason for this discrepancy between adults and children was also unknown, with the authors offering a possibility relating to body size.

Our paper describes in vitro coagulant activity of venoms upon plasma, which has been shown to correlate with in vivo defibrinogenating effects [72]. However, it is important to note that venom-induced differences have also been shown between paediatric and adult whole blood. For example, platelet aggregation was inhibited by *N. scutatus* venom more so in paediatric blood than in adult blood [73]. This difference was not observed with *Pseudonaja textilis* (Eastern Brown Snake) venom. It is important to note that, while blood type has been shown to have minor effects on coagulation clotting times [74], our results do not suggest that ABO blood type of our plasmas (i.e., AB+ for adult; unknown for paediatric plasma but highly likely a mixture due to 21 samples being pooled) impacted the results. If the blood type did affect the observed clotting times, we would expect to observe a similar pattern of difference b/w adult and paediatric plasma across the different snake venoms, yet this was not the case; we observed almost parity between the two plasma types for most venoms, except for a few venoms which is discussed above.

While in vitro tests in the lab are informative comparisons in all-else-being-equal scenario, many factors besides the venom toxicity (i.e., drop-for-drop activity levels) can greatly influence the extent and speed of clinical symptoms and therefore the medical outcome of an envenomation [75]. Factors that likely influence clinical envenomations include (but are not limited to): venom dose, fang length, bite site location on the body, snakebite first-aid used (e.g., compression bandage, remaining still), the species envenomated, and body size of the victim. Indeed, given that only three out of 15 venoms affected paediatric plasma significantly differently than adult plasma, these factors probably have a much greater influence on the severity of patient coagulopathy than the venom components and mechanisms alone. 

This point is clearly illustrated in this example: an 80 kg adult is estimated to have around 5.6 L of blood, while a 14.5 kg child (3 years old) has around 1.16 L of blood, or 21% that of adults [76]. As such, once venom entered the bloodstream, it would theoretically be around five times more concentrated in a child than in an adult, merely due to body size. This factor is expected to greatly affect the severity of envenomation, as well as possibly determine the net effect of a procoagulant venom. For example, envenomated prey items such as rats are likely to suffer a stroke from blood vessel occlusion (blockage) [34,35,36,37], but adult humans often succumb instead to venom-induced consumptive coagulopathy (VICC) [38,77,78] due to many micro-clots being formed that are not large enough to cause blood vessel occlusion but still consume blood clotting factors. 

### Conclusions

The blood circulation system is critical for vertebrate functioning because it maintains haemostasis and distributes nutrients, hormones, electrolytes, immune system cells, and gases throughout the body. When this system is disrupted (e.g., by disease or toxins), devastating effects or even death can occur. Our preliminary study provides new insights into possible differences between adult and paediatric plasma and their effect on snake venom function. While no in vitro test can truly measure the overall functionality of the haemostatic system, our tests provide an all-else-being-equal comparison of the pathophysiological susceptibility of paediatric and adult plasmas to coagulotoxic snake venoms of medical significance. We hope our research informs future studies on the effects of snake venom on paediatric plasma to reduce the burden of snakebite upon society.

## 3. Materials and Methods

### 3.1. Plasma Collection and Handling

Citrated, platelet-poor plasma from two demographics were collected for testing: adult and child. Surplus platelet-poor plasma (292 mL) from an unknown number of healthy adults (18+ years old; AB+; Label #4731976) was provided by the Australian Red Cross, under Research Agreement #18-03QLD-09. The plasma bag (292 mL) was defrosted, pooled, aliquoted, flash-frozen in liquid nitrogen, and stored at −80 °C until required, as per previously established protocols [51]. The same pooling and freezing procedures were carried out for paediatric plasma. 

Paediatric platelet-poor plasma was collected from 22 children aged 1.3–4.9 years old (median 3.3; 13 males and 9 females) (unknown blood types) attending the Royal Children’s Hospital, Melbourne for minor day surgery who did not have a family history of coagulation disorders (e.g., thrombosis). This is the only paediatric plasma to which we had access. Family history was assessed via a brief interview with the parents of the children. This study was approved by the Royal Children’s Hospital Ethics in Human Research Committee (#20031). Informed consent was obtained from the parents of the children. All blood samples were collected and stored in one volume of 0.106 mol L^−1^ citrate per nine volumes of blood. Samples were then centrifuged at 1450× *g* for 10 min at 10 °C (Megafuge 1.0R; Heraeus, Thermo Scientific, Karlsruhe, Germany), and platelet-poor plasma was stored at −80 °C until testing.

For testing, one plasma aliquot at a time was thawed for five minutes at 37 °C in a Thermo Haake ARCTIC water bath with a SC150-A40 circulator. Defrosted plasma aliquots were replaced every 30 min at maximum to maintain freshness. 

All plasma work was undertaken under University of Queensland Biosafety Approval #IBC134BSBS2015 and University of Queensland Human Ethics Committee Approval #2016000256. 

### 3.2. Venom Stocks

All venom and plasma work were undertaken under the UQ approval IBC134BSBS2015. Working stocks of venom were made to 1 mg/mL concentration with 50% glycerol to prevent freezing at −20°C. Venom protein concentration was determined at 280 nm with a Thermo Fisher Scientific™ NanoDrop 2000 UV–Vis Spectrophotometer (Thermofisher, Sydney, NSW, Australia). To control for individual variation in venoms when comparing across species [79,80,81], for all except one venom (*A. bibronii*) we were able to make/use pooled samples from 2–8 individuals per venom sample. Samples were pooled either during venom extraction (snakes bit the same vial), or in the lab. To pool individually extracted venom samples ex post facto, we first made the stocks to 1 mg/mL and then transferred equal volumes from each sample into a single, new tube. Venoms included in the study are listed in Table 3.

### 3.3. Coagulation Tests

Clotting times (seconds) of plasma were automatically measured using a STA-R Max^®^ analyser (Stago, Asnières sur Seine, France). Measurements occurred via a viscosity-based (mechanical) detection system, whereby opposing magnets oscillate a metal pellet inside the test cuvette (250 µL total volume) until a clot is formed. 

To establish baseline clotting time values of our two plasma batches, three standard coagulation assays (Table 2) were performed on both plasma types in the absence of venom. PT is a coagulation screening test which measures, as a whole, activity of the extrinsic and common pathway, including activity of the coagulation factors VII, X, V, II, and fibrinogen. Another test, aPTT, is a general coagulation screening test of the intrinsic pathway, including the coagulation factors XII, XI, IX, VIII, X, V, II, and fibrinogen. 

Venom-induced clotting times were used as a measure of susceptibility to the coagulotoxic nature of the venoms included. To test procoagulant and pseudo-procoagulant venoms (the latter makes weak, transient clots [82]), we used an aPTT-based assay (‘Procoag. venom’, Table 2) to accommodate venom in the assay and remove the clotting catalyst (Kaolin) in the standard aPTT assay so as to not mask the venom action. A separate assay (‘Anticoag. venom’, Table 2), which uses an FX reagent to attempt to clot the plasma after the venom incubates with the plasma for 120 s, was used to test the potency of anticoagulant venoms on the plasmas. For all dose-response curves, dilution of the venoms with OK buffer was performed automatically by the machine. The venom dilution series was as follows: 1:2, 1:5, 1:12, 1:30, 1:80, 1:160, and 1:400, yielding final cuvette concentrations of venom ranging from 0.05 µg/mL to 20 μg/mL. The most dilute concentration (1:400) was replaced by 1:200 for the thrombin and FXa assays due to dilution constraints of the assay. Specific assay details are listed in Table 2. Venoms were run on either the procoagulant or anticoagulant assay based on our previous work (see Table 3 for all venoms and activities) that determined the venoms were procoagulant or anticoagulant in nature on human plasma.

The experimental workflow went as follows: A quality control (aPTT [83] on adult plasma) was run at the start of each day to ensure the plasma was functioning properly and as per all other experimental days. A new venom solution was made up for each concentration curve replicate, with each dilution-point replicate of the triplicate being run independently of each other. Each venom was run on both plasma types before testing the next venom, ensuring the fairest comparison between plasma types across all venoms. 

Reagents were kept at 15–19 °C in the machine during experimentation and otherwise stored at 4 °C. Venom samples were replaced at maximum every 15 min to minimise degradation. Since venoms were made up in 50% glycerol, the blank replacement for venom in the negative control tests was 50% glycerol. All tests were performed in triplicate. The same analyser and reagents were used for all experiments, thereby making the results herein comparable.

### 3.4. Statistical Analyses

All stats tests were performed using GraphPad Prism software (v.7.0). We did not test for normality of data due to only three replicates being present per test. To be conservative, for the standard coagulation assays, Kolmogorov–Smirnov tests were used to test significance using cumulative distributions (rather than ranks). 

Area Under the Curve (AUC) values of venom-induced clotting time curves were calculated separately for each curve replicate. The Standard Deviation (SD) values were calculated using the three AUC replicates for each curve. To determine if significant differences between plasma types occurred within each venom, multiple *t*-test comparisons were made between the three adult AUC values and the three paediatric AUC values for each venom. Although AUC values were derived from a series of 8-pt curves run in triplicate, we still only had three replicates of AUC values. Due to this small sample size of AUC values (N = 3), normality was not tested. Instead, a False Discovery Rate (FDR) approach was used, with a two-stage step-up method of Benjamini and Yekutieli [84]. Since we were interested in major, clear differences between the two plasma types, a desired FDR (Q) of 1% was chosen. Q is the proportion of false discoveries divided by the total number of discoveries. A 1% cut-off is more robust than a 5% value because this value determines the acceptable percentage of discoveries that will prove to be false. In other words, no more than 1% of significant ‘discoveries’ will be false discoveries (due to random scatter of data) while at least 99% of the discoveries are true differences between the AUC means. Because each row (venom) represented different measures, each row was analysed independently to not assume consistent SD. Although this analysis reduced the degrees of freedom and thus power, it enabled robust analyses with fewer assumptions.

## Figures and Tables

**Table 1:** Snake venoms included and their known modes of coagulotoxic action.

Scientific Name	Common Name	Broad Distribution	Coagulotoxic Activity and Mechanism	Source of Mechanism
*Atractaspis bibronii*	Bibron’s Stiletto Snake	Southern Africa	FX activating	[41]
*Bitis cornuta*	Many-horned Adder	Atlantic coast of South Africa	Prothrombinase inhibition via PLA_2_s	[42]
*Bothrops atrox*	Lancehead Viper	South America	FX and PRT activating via SVMP	[43]
*Bothrops asper*	Terciopelo	Central America	FX and PRT activating via SVMP	[36]
*Crotalus mictlantecuhtli*	Veracruz Neotropical Rattlesnake	Mexico	Fibrinogen cleavage (pseudo-procoagulant)	[44]
*Daboia russelii*	Russell’s Viper	South Asia	FX activating	[45]
*Dispholidus typus*	Boomslang	Sub-Saharan Africa	PRT activating	[46]
*Echis coloratus*	Painted Saw-scaled Viper	Middle East and Arabian Peninsula	PRT activating via P-IIId SVMP (Ca-dependent)	[47]
*Echis ocellatus*	West African Saw-scaled Viper	West Africa	PRT activating via P-IIIa SVMP (Ca-independent)	[48]
*Macrovipera lebetina cernovi*	Lebetine Viper	Middle East	FX activating	[49]
*Notechis scutatus*	Tiger Snake	South and SE Australia	PRT activating via venom FXa	[50]
*Pseudechis australis*	Mulga Snake	South and SE Australia	Prothrombinase and/or FVa inhibition via PLA_2_s	[51]
*Pseudonaja textilis*	Eastern Brown Snake	Eastern & Southern Australia	PRT activating via venom FVa:FXa	[52]
*Thelotornis mossambicanus*	Eastern Twig Snake	Eastern Africa	PRT activating	[46]
*Trimeresurus albolabris*	White-lipped Pit Viper	India and SE Asia	Fibrinogen cleavage (pseudo-procoagulant)	[53,54]

F = factor; PRT = Prothrombin; Ca = Calcium; SVMP = Snake Venom Metalloproteinase; PLA_2_s = Phospholipase A_2_s.

**Table 2:** Area Under the Curve ± SD values, derived from 8-point dilution curves of venom added to either paediatric or adult human plasma (as per methods in Table 2, and *t*-test q-values for 1% False Discovery Rate using two-stage step-up method.

Species	Common Name	Adult Plasma	Paediatric Plasma	*t*-Test * q-Value	*t* Ratio
*Atractaspis bibronii*	Bibron’s stiletto snake	536.3 ± 29.2	516.1 ± 5.6	q = 0.43	1.18
*Bitis cornuta*	Many-horned adder	1794.0 ± 25.3	940.6 ± 33.1	**q < 0.0001**	35.43
*Bothrops asper*	Terciopelo	272.7 ± 2.5	270.0 ± 8.0	q = 0.53	0.56
*Bothrops atrox*	Fur-de-lance	458.2 ± 21.5	401.1 ± 5.0	q = 0.08	4.49
*Crotalus mictlantecuhtli*	Veracruz Neotropical rattlesnake	2962.0 ± 33.4	2924.0 ± 126.8	q = 0.53	0.05
*Daboia russelii*	Russell’s viper	202.0 ± 1.5	154.1 ± 2.8	**q < 0.001**	26.21
*Dispholidus typus*	Boomslang	434.2 ± 19.8	462.8 ± 12.1	q = 0.20	2.14
*Echis coloratus*	Painted saw-scaled viper	591.1 ± 6.6	568.3 ± 19.6	q = 0.26	1.90
*Echis ocellatus*	West African carpet viper	356.1 ± 22.6	366.1 ± 22.7	q = 0.53	0.54
*Macrovipera lebentina cernovi*	Lebetine viper	167.1 ± 2.8	154.3 ± 2.6	q = 0.01	5.77
*Notechis scutatus*	Tiger snake	124.6 ± 1.9	120.3 ± 9.0	q = 0.53	0.81
*Pseudechis australis*	Mulga snake	2142.0 ± 28.1	342.8 ± 9.9	**q < 0.0001**	104.6
*Pseudonaja textilis*	Eastern brown snake	67.7 ± 1.6	68.3 ± 0.98	q = 0.53	0.58
*Thelotornis mossambicanus*	Eastern twig snake	204.4 ± 4.4	220.5 ± 3.2	q = 0.02	5.13
*Trimeresurus albolabris*	White-lipped pit viper	2158.0 ± 33.5	2045.0 ± 133.5	q = 0.38	1.41

* Unpaired, parametric *t*-tests using 1% False Discovery Rate threshold for significance, comparing the Area Under the Curve values of adult vs. paediatric plasma Note: bolded *p*-values indicate ‘true’ differences according to the 1% False Discovery Rate.

**Table 3:** Coagulation assays used to compare adult and paediatric plasma *.

Assay	Methodology
Standard coagulation tests	
aPTT	Step 1: 50 µL plasma + 50 µL kaolin/phospholipid (Stago # 00597) Step 2: 240 s incubation at 37 °C Step 3: Addition of 50 µL 0.025 M calcium (Stago # 00367)
PT	Step 1: 50 µL human plasma Step 2: 240 s incubation at 37 °C Step 3: Addition of 100 µL Neoplastine (Stago #00606)
Fibrinogen levels	Step 1: 150 µL plasma (diluted 1:20 by Owren–Koller (OK) Buffer (isotonic saline) (Stago # 00360) Step 2: 240 s incubation at 37 °C Step 3: Addition of 50 µL Thrombin (Stago #00611)
Venom tests	
Procoag. venom	Step 1: 50 µL 0.1 µg/mL venom (diluted in OK for dose-response curves) + 50 µL 0.025 M calcium + 50 µL phospholipid (phospholipid bottle in Stago kit #00597) + 25 µL OK buffer Step 2: 120 s incubation at 37 °C Step 3: Addition of 75 µL plasma
Anticoag. venom	Step 1: 25 µL 0.2 µg/mL venom (diluted in OK for dose-response curves) + 50 µL 0.025 M calcium + 50 µL phospholipid + 25 µL OK Buffer + 75 µL plasma Step 2: 120 s incubation at 37 °C Step 3: Addition of 25 µL FXa (FXa bottle in Stago kit # 00311)

* Negative control conditions for ‘Venom tests’ replaced venom with a blank. aPTT = Activated Partial Thromboplastin Time; PT = Prothrombin Time; Procoag. = procoagulant; ‘Anticoag.’ = anticoagulant.

## Data Availability

Data are available upon request to the authors.

## References

[1] Kasturiratne A., Wickremasinghe A.R., De Silva N., Gunawardena N.K. (2008). The Global Burden of Snakebite: A Literature Analysis and Modelling Based on Regional Estimates of Envenoming and Deaths. PLoS Med..

[2] Harrison R.A., Hargreaves A., Wagstaff S.C., Faragher B., Lalloo D.G. (2009). Snake Envenoming: A Disease of Poverty. PLoS Negl. Trop. Dis..

[3] Mohapatra B., Warrell D.A., Suraweera W., Bhatia P., Dhingra N., Jotkar R.M., Rodriguez P.S., Mishra K., Whitaker R., Jha P. (2011). Snakebite Mortality in India: A Nationally Representative Mortality Survey. PLoS Negl. Trop. Dis..

[4] Schiermeier Q. Snakebite Crisis Gets US$100-Million Boost for Better Antivenoms: Wellcome Trust Launches Research Initiative for Long-Neglected Health Problem. Nat. News.

[5] Gutiérrez J.M., Solano G., Pla D., Herrara M., Segura A., Vargas M., Villalta M., Sanchez A., Sanz L., Lomonte B. (2017). Preclinical Evaluation of the Efficacy of Antivenoms for Snakebite Envenoming: State-of-the-Art and Challenges Ahead. Toxins.

[6] Gutiérrez J.M., Burnouf T., Harrison R., Calvete J., Kuch U., Warrell A. (2014). A Multicomponent Strategy to Improve the Availability of Antivenom for Treating Snakebite Envenoming. Policy Pract..

[7] Fry B.G. (2015). Venomous Reptiles and Their Toxins: Evolution, Pathophysiology, and Biodiscovery.

[8] Gutiérrez J.M., Calvete J.J., Habib A.G., Harrison R.A., Williams D.J., Warrell D.A. (2017). Snakebite Envenoming. Nat. Rev. Dis. Prim..

[9] Clarke C., Kuruppu S., Reeve S., Ian Smith A., Hodgson W.C. (2006). Oxylepitoxin-1, a Reversible Neurotoxin from the Venom of the Inland Taipan (*Oxyuranus Microlepidotus*). Peptides.

[10] Isbister G.K., Duffull S.B., Brown S.G.A. (2009). Failure of Antivenom to Improve Recovery in Australian Snakebite Coagulopathy. QJM An Int. J. Med..

[11] Palta S., Saroa R., Palta A. (2014). Overview of the Coagulation System. Indian J. Anaesth..

[12] Casewell N.R., Jackson T.N.W., Laustsen A.H., Sunagar K. (2020). Causes and Consequences of Snake Venom Variation. Trends Pharmacol. Sci..

[13] Vonk F.J., Casewell N.R., Henkel C.V., Heimberg A.M., Jansen H.J., McCleary R.J.R., Kerkkamp H.M.E., Vos R.A., Guerreiro I., Calvete J.J. (2013). The King Cobra Genome Reveals Dynamic Gene Evolution and Adaptation in the Snake Venom System. Proc. Natl. Acad. Sci. USA.

[14] Bos M.H.A., Boltz M., St. Pierre L., Masci P.P., De Jersey J., Lavin M.F., Camire R.M. (2009). Venom Factor V from the Common Brown Snake Escapes Hemostatic Regulation through Procoagulant Adaptations. Blood.

[15] Reza M.A., Le T.N.M., Swarup S., Kini R.M. (2006). Molecular Evolution Caught in Action: Gene Duplication and Evolution of Molecular Isoforms of Prothrombin Activators in Pseudonaja Textilis (Brown Snake). J. Thromb. Haemost..

[16] Andrew M., Paes B., Johnston M. (1990). Development of the Hemostatic System in the Neonate and Young Infant. Am. J. Pediatr. Hematol. Oncol..

[17] Parrish M., Goldner C., Silberg L. (1965). Comparison between Snakebites in Children and Adults. Pediatrics.

[18] Wood D., Sartorius B., Hift R. (2016). Snakebite in North-Eastern South Africa: Clinical Characteristics and Risks for Severity. South African Fam. Pract..

[19] Smith H., Brown D. (2019). Multiple Thromboembolic Strokes in a Toddler Associated with Australian Eastern Brown Snake Envenomation. Radiol. Case Reports.

[20] Brenes-Chacón H., Gutiérrez J.M., Camacho-Badilla K., Soriano-Fallas A., Ulloa-Gutierrez R., Valverde-Muñoz K., Ávila-Agüero M.L. (2019). Snakebite Envenoming in Children: A Neglected Tropical Disease in a Costa Rican Pediatric Tertiary Care Center. Acta Trop..

[21] Variawa S., Buitendag J., Marais R., Wood D., Oosthuizen G. (2021). Prospective Review of Cytotoxic Snakebite Envenomation in a Paediatric Population. Toxicon.

[22] Buitendag J., Variawad S., Wood D., Oosthuizen G.V. (2022). A Comparison between Adult and Paediatric Snakebites and Their Outcomes in North Eastern South Africa. Toxicon.

[23] Tekin R., Sula B., Cakir G., Aktar F., Deveci, Yolbas I., Çelen M.K., Bekcibasi M., Palanci Y., Dogan E. (2015). Comparison of Snakebite Cases in Children and Adults. Eur. Rev. Med. Pharmacol. Sci..

[24] Levine M., Ruha A.M., Wolk B., Caravati M., Brent J., Campleman S., Wax P., Aldy K., Akpunonu P., Bebarta V.S. (2020). When It Comes to Snakebites, Kids Are Little Adults: A Comparison of Adults and Children with Rattlesnake Bites. J. Med. Toxicol..

[25] Suryanarayana G., Rameshkumar R., Mahadevan S. (2021). Retrospective Hospital-Based Cohort Study on Risk Factors of Poor Outcome in Pediatric Snake Envenomation. J. Trop. Pediatr..

[26] Monagle P., Barnes C., Ignjatovic V., Furmedge J., Newall F., Chan A., De Rosa L., Hamilton S., Ragg P., Robinson S. (2006). Developmental Haemostasis: Impact for Clinical Haemostasis Laboratories. Thromb. Haemost..

[27] Ignjatovic V., Pelkmans L., Kelchtermans H., Al Dieri R., Hemker C., Kremers R., Bloemen S., Karlaftis V., Attard C., De Laat B. (2015). Differences in the Mechanism of Blood Clot Formation and Nanostructure in Infants and Children Compared with Adults. Thromb. Res..

[28] Attard C., Van der Straaten T., Karlaftis V., Monagle P., Ignjatovic V. (2013). Developmental Hemostasis: Age-Specific Differences in the Levels of Hemostatic Proteins. J. Thromb. Haemost..

[29] Kremers R.M.W., Wagenvoord R.J., de Laat H.B., Monagle P., Hemker H.C., Ignjatovic V. (2016). Low Paediatric Thrombin Generation Is Caused by an Attenuation of Prothrombin Conversion. Thromb. Haemost..

[30] Roeloffzen W.W., Kluin-Nelemans H.C., Mulder A.B., Veeger N.J.G.M., Bosman L., De Wolf J.T. (2010). In Normal Controls, Both Age and Gender Affect Coagulability as Measured by Thrombelastography. Anesth. Analg..

[31] Mosquera A., Idrovo L.A., Tafur A., Del Brutto O.H. (2003). Stroke Following *Bothrops* Spp. Snakebite. Neurology.

[32] Silva de Oliveira S., Freitas-de-Sousa L.A., Alves E.C., de Lima Ferreira L.C., da Silva I.M., de Lacerda M.V.G., Fan H.W., Moura-da-Silva A.M., Monteiro W.M. (2017). Fatal Stroke after Bothrops Snakebite in the Amazonas State, Brazil: A Case Report. Toxicon.

[33] de Oliveira Pardal P.P., da Silva Pinheiro A.C.J., Cunha Silva C.T., Santos P.R.S.G., da Costa Gadelha M.A. (2015). Hemorrhagic Stroke in Children Caused by *Bothrops Marajoensis* Envenoming: A Case Report. J. Venom. Anim. Toxins Incl. Trop. Dis..

[34] Tian H., Liu M., Li J., Xu R., Long C., Li H., Mwangi J., Lu Q., Lai R., Shen C. (2020). Snake C-Type Lectins Potentially Contribute to the Prey Immobilization in *Protobothrops mucrosquamatus* and *Trimeresurus stejnegeri* Venoms. Toxins.

[35] Herrera M., Fernández J., Vargas M., Villalta M., Segura Á., León G., Angulo Y., Paiva O., Matainaho T., Jensen S.D. (2012). Comparative Proteomic Analysis of the Venom of the Taipan Snake, *Oxyuranus Scutellatus*, from Papua New Guinea and Australia: Role of Neurotoxic and Procoagulant Effects in Venom Toxicity. J. Proteomics.

[36] Loría G.D., Rucavado A., Kamiguti A.S., Theakston R.D.G., Fox J.W., Alape A., Gutiérrez J.M. (2003). Characterization of “basparin A,” a Prothrombin-Activating Metalloproteinase, from the Venom of the Snake *Bothrops Asper* That Inhibits Platelet Aggregation and Induces Defibrination and Thrombosis. Arch. Biochem. Biophys..

[37] Martin C.J. (1893). On Some Effects upon the Blood Produced by the Injection of the Venom of the Australian Black Snake (*Pseudechis Porphyriacus*). Proc. R. Soc. New South Wales.

[38] Isbister G.K., Scorgie F.E., O’leary M.A., Seldon M., Brown S.G.A., Lincz L.F. (2010). Factor Deficiencies in Venom-Induced Consumption Coagulopathy Resulting from Australian Elapid Envenomation: Australian Snakebite Project (ASP-10). J. Thromb. Haemost..

[39] Monagle P., Ignjatovic V., Savoia H. (2010). Hemostasis in Neonates and Children: Pitfalls and Dilemmas. Blood Rev..

[40] Lee J., Kim J. (2022). Hemostatic Analysis of Simulated *Gloydius Ussuriensis* Envenomation Using Canine Blood: A Comparison of Thromboelastography and Classical Coagulation Tests. Animals.

[41] Oulion B., Dobson J.S., Zdenek C.N., Arbuckle K., Lister C., Coimbra F.C.P., op den Brouw B., Debono J., Rogalski A., Violette A. (2018). Factor X Activating Atractaspis Snake Venoms and the Relative Coagulotoxicity Neutralising Efficacy of African Antivenoms. Toxicol. Lett..

[42] Youngman N.J., Walker A., Naude A., Coster K., Sundman E., Fry B.G. (2020). Varespladib (LY315920) Neutralises Phospholipase A2 Mediated Prothrombinase-Inhibition Induced by Bitis Snake Venoms. Comp. Biochem. Physiol. Part C Toxicol. Pharmacol..

[43] Sousa L., Zdenek C.N., Dobson J., op den Brouw B., Coimbra F., Gillett A., Del-Rei T., Chalkidis H., Sant’Anna S., Teixeira-da-Rocha M. (2018). Coagulotoxicity of *Bothrops* (Lancehead Pit-Vipers) Venoms from Brazil: Differential Biochemistry and Antivenom Efficacy Resulting from Prey-Driven Venom Variation. Toxins.

[44] Seneci L., Zdenek C.N., Bourke L.A., Cochran C., Elda E.S., Frank N., Fry B.G., Neri-castro E., Melisa B., Alag A. (2021). A Symphony of Destruction: Dynamic Differential Fibrinogenolytic Toxicity by Rattlesnake (*Crotalus* and *Sistrurus*) Venoms. Comp. Biochem. Physiol. A Mol. Integr. Physiol..

[45] Zhu W., Wu Z., Shen S., Liu J., Xiang N., Liao Y., Lin X., Chen L., Chen Q. (2015). Purification, Partial Characterizations, and N-Terminal Amino Acid Sequence of a Procoagulant Protein FV-2 from *Daboia Russelli Siamensis* (Myanmar) Venom. J Biochem Mol. Toxicol..

[46] Debono J., Dashevsky D., Nouwens A., Fry B.G. (2020). The Sweet Side of Venom: Glycosylated Prothrombin Activating Metalloproteases from *Dispholidus typus* (Boomslang) and *Thelotornis mossambicanus* (Twig Snake). Comp. Biochem. Physiol. Part C Toxicol. Pharmacol..

[47] Morita T., Iwanaga S. (1978). Purification and Properties of Prothrombin Activator from the Venom of *Echis Carinatus*. J. Biochem..

[48] Hasson S.S., Theakston R.D.G., Harrison R.A. (2003). Cloning of a Prothrombin Activator-like Metalloproteinase from the West African Saw-Scaled Viper, *Echis Ocellatus*. Toxicon.

[49] Chowdhury A., Zdenek C.N., Dobson J.S., Bourke L.A., Soria R., Fry B.G. (2021). Clinical Implications of Differential Procoagulant Toxicity of the Palearctic Viperid Genus *Macrovipera*, and the Relative Neutralization Efficacy of Antivenoms and Enzyme Inhibitors. Toxicol. Lett..

[50] Kini R.M. (2005). The Intriguing World of Prothrombin Activators from Snake Venom. Toxicon.

[51] Zdenek C.N., Youngman N.J., Hay C., Dobson J., Dunstan N., Allen L., Milanovic L., Fry B.G. (2020). Anticoagulant Activity of Black Snake (Elapidae: *Pseudechis*) Venoms: Mechanisms, Potency, and Antivenom Efficacy. Toxicol. Lett..

[52] Masci P.P., Whitaker A.N., de Jersey J. (1988). Purification and Characterization of a Prothrombin Activator from the Venom of the Australian Brown Snake, *Pseudonaja Textilis Textilis*. Biochem Int..

[53] Bourke L.A., Youngman N.J., Zdenek C.N., op den Brouw B., Violette A., Fourmy R., Fry B.G. (2020). *Trimeresurus Albolabris* Snakebite Treatment Implications Arising from Ontogenetic Venom Comparisons of Anticoagulant Function, and Antivenom Efficacy. Toxicol. Lett..

[54] Debono J., Bos M.H.A., Frank N., Fry B. (2019). Clinical Implications of Differential Antivenom Efficacy in Neutralising Coagulotoxicity Produced by Venoms from Species within the Arboreal Viperid Snake Genus *Trimeresurus*. Toxicol. Lett..

[55] Ignjatovic V., Ilhan A., Monagle P. (2011). Evidence for Age-Related Differences in Human Fibrinogen. Blood Coagul. Fibrinolysis.

[56] Clauss A. (1957). Rapid Physiological Coagulation Method in Determination of Fibrinogen. Acta Haematol..

[57] Warrell D.A. (1989). Snake Venoms in Science and Clinical Medicine * 1. Russell’s Viper: Biology, Venom and Treatment of Bites. Trans R Soc Trop Med Hyg.

[58] Alirol E., Lechevalier P., Zamatto F., Chappuis F., Alcoba G., Potet J. (2015). Antivenoms for Snakebite Envenoming: What Is in the Research Pipeline?. PLoS Negl. Trop. Dis..

[59] Glaudas X. (2021). Natural History of a Highly Medically Important Snake, Russell’s Viper (*Daboia Russelii*), in a Human-Dominated Indian Rural Landscape. J. Herpetol..

[60] Dissanayake P.V., Muthukumarana T.G.W., Aslam W.A.M., Chaminda S.A.A., Munasinghe T.S., Kularatne S.A.M. (2019). An Unusual Case of Gross Myoglobinuria in a Child Following Russell’s Viper (*Daboia Russelii*) Envenomation. Toxicon.

[61] Mukherjee A.K. (2014). The Pro-Coagulant Fibrinogenolytic Serine Protease Isoenzymes Purified *from Daboia Russelii* Russelii Venom Coagulate the Blood through Factor V Activation: Role of Glycosylation on Enzymatic Activity. PLoS ONE.

[62] Sharma M., Das D., Iyer J.K., Kini R.M., Doley R. (2015). Unveiling the Complexities of *Daboia Russelii* Venom, a Medically Important Snake of India, by Tandem Mass Spectrometry. Toxicon.

[63] Kini R.M., Rao V.S., Joseph,  J.S. (2002). Procoagulant Proteins from Snake Venoms. Haemostasis.

[64] Andrew M., Vegh P., Johnston M., Bowker J., Ofosu F., Mitchell L. (1992). Maturation of the Hemostatic System during Childhood. Blood.

[65] Han K.K., Martinage A. (1992). Post-Translational Chemical Modification(S) of Proteins. Int. J. Biochem..

[66] Bevers E.M., Williamson P.L. (2016). Getting to the Outer Leaflet: Physiology of Phosphatidylserine Exposure at the Plasma Membrane. Physiol. Rev..

[67] Zdenek⁠ C.N., Hay C., Arbuckle K., Jackson T.N.W., Bos M.H.A., op den Brouw B., Debono J., Allen L., Dunstan N., Morley T. (2019). Coagulotoxic Effects by Brown Snake (*Pseudonaja*) and Taipan (*Oxyuranus*) Venoms, and the Efficacy of a New Antivenom. Toxicol. Vitr..

[68] Bernhard H., Rosenkranz A., Novak M., Leschnik B., Petritsch M., Rehak T., Köfeler H., Ulrich D., Muntean W. (2009). No Differences in Support of Thrombin Generation by Neonatal or Adult Platelets. Hamostaseologie.

[69] Youngman N.J., Debono J., Dobson J.S., Zdenek C.N., Harris R.J., den Brouw B.O., Coimbra F.C.P., Naude A., Coster K., Sundman E. (2019). Venomous Landmines: Clinical Implications of Extreme Coagulotoxic Diversification and Differential Neutralization by Antivenom of Venoms within the Viperid Snake Genus Bitis. Toxins.

[70] Andrew M., Paes B., Milner R., Johnston M., Mitchell L., Tollefsen D., Castle V., Powers P. (1988). Development of the Human Coagulation System in the Healthy Premature Infant. Blood.

[71] St. Pierre L., Masci P.P., Filippovich I., Sorokina N., Marsh N., Miller D.J., Lavin M.F. (2005). Comparative Analysis of Prothrombin Activators from the Venom of Australian Elapids. Mol. Biol. Evol..

[72] Gutiérrez J.M., Vargas M., Segura A., Herrera M., Villalta M., Solano G., Sánchez A., Herrera C., León G. (2021). In Vitro Tests for Assessing the Neutralizing Ability of Snake Antivenoms: Toward the 3Rs Principles. Front. Immunol..

[73] Kern L.P., Ignjatovic V., Winkel K.D., Summerhayes R., Monagle P. (2008). The Differences of Platelet Response to Snake Venoms: A Comparative Study of Children and Adults. Toxicon.

[74] Choi Q., Kim J.E., Kim S.Y., Han K.S., Kim H.K. (2015). Influence of ABO Type on Global Coagulation Assay Results: Effect of Coagulation Factor VIII. Clin. Chem. Lab. Med..

[75] Underhill D., Sutherland S.K. (1993). Snake Species. Australia’s Dangerous Creatures.

[76] Howie S.R.C. (2011). Blood Sample Volumes in Child Health Research: Review of Safe Limits. Bull. World Health Organ..

[77] Maduwage K., Isbister G.K. (2014). Current Treatment for Venom-Induced Consumption Coagulopathy Resulting from Snakebite. PLoS Negl. Trop. Dis..

[78] Isbister G.K. (2010). Snakebite Doesn’t Cause Disseminated Intravascular Coagulation: Coagulopathy and Thrombotic Microangiopathy in Snake Envenoming. Semin Thromb Hemost.

[79] Laxme R.R.S., Khochare S., Attarde S., Suranse V., Iyer A., Casewell N.R., Whitaker R., Martin G., Sunagar K. (2021). Biogeographic Venom Variation in Russell’s Viper (*Daboia Russelii*) and the Preclinical Inefficacy of Antivenom Therapy in Snakebite Hotspots. PLoS Negl. Trop. Dis..

[80] Children A., Adelaide N., Museum S.A., Terrace N., Terrace N. (1988). Variation in Venom Proteins from Isolated Populations of Tiger Snakes (*Notechis Ater Niger, N. Scutatus*) in South Australia. Toxicon.

[81] Flight S., Mirtschin P., Masci P.P. (2006). Comparison of Active Venom Components between Eastern Brown Snakes Collected from South Australia and Queensland. Ecotoxicology.

[82] Bourke L.A., Zdenek C.N., Tanaka-azevedo A.M., Perez G., Silveira M., Sant S., Grego K.F., Fabri C., Rodrigues B., Fry B.G. (2022). Clinical and Evolutionary Implications of Dynamic Coagulotoxicity Divergences in *Bothrops* (Lancehead Pit Viper) Venoms. Toxins.

[83] Langdell R., Wagner R., Brinkhous K. (1953). Effect of Antihemophilic Factor on One-Stage Clotting Tests. J. Lab. Clin. Med..

[84] Benjamini Y., Yekutieli D. (2005). Quantitative Trait Loci Analysis Using the False Discovery Rate. Genetics.

